# Acceptability of alginate enriched bread and its effect on fat digestion in humans

**DOI:** 10.1016/j.foodhyd.2019.02.027

**Published:** 2019-08

**Authors:** David Houghton, Matthew D. Wilcox, Iain A. Brownlee, Peter I. Chater, Chris J. Seal, Jeffrey P. Pearson

**Affiliations:** a Institute for Cell and Molecular Biosciences, Newcastle University, Medical School, Framlington Place, Newcastle upon Tyne, UK; b Human Nutrition Research Centre, Institute of Cellular Medicine, Newcastle University, M2.054 Leech Building, Newcastle upon Tyne, NE2 4HH, UK

**Keywords:** Alginate, Fat digestion, Acceptability, Obesity, Lipase inhibition, Lipolysis

## Abstract

Lifestyle interventions and physical activity remain the cornerstone of obesity management, as pharmacological therapies (orlistat) are associated with gastrointestinal (GI) side effects. Combining orlistat with fibers can reduce side effects, improving compliance. Therefore, a fiber that inhibits lipase without side effects could help treat obesity.

The aims of the present work were to assess whether alginate enriched bread could inhibit fat digestion, and assess the acceptability of alginate bread and its effect on GI wellbeing.

A double-blind, randomised, controlled cross-over pilot study (NCT03350958) assessed the impact of an alginate bread meal on; lipid content in ileal effluent and circulating triacylglycerol levels. This was compared against the same meal with non-enriched (control) bread.

GI wellbeing and acceptability of alginate bread was compared to control bread through daily wellbeing questionnaires and food diaries (NCT03477981). Control bread followed by alginate bread were consumed for two weeks respectively.

Consumption of alginate bread reduced circulating triacylglycerol compared to control (2% reduction in AUC) and significantly increased lipid content in ileal effluent (3.8 g ± 1.6 after 210 min).

There were no significant changes to GI wellbeing when comparing alginate bread to control bread. A significant increase in the feeling of fullness occurred with alginate bread compared to baseline and the first week of control bread consumption.

This study showed that sustained consumption of alginate enriched bread does not alter GI wellbeing and can decrease lipolysis, increasing lipid leaving the small intestine.

Further studies are required to demonstrate that reduced fat digestion through the action of alginate can reduce fat mass or body weight.

## Highlights

• Alginate can be incorporated into a highly acceptable loaf at 4%.
• Sustained (two weeks) consumption of alginate bread did not affect GI wellbeing.
• Consumption of alginate bread decreases circulating triglyceride after the meal.
• Consumption of alginate bread increases lipid leaving the ileum after the meal.

## Graphical abstract

**Figure:**
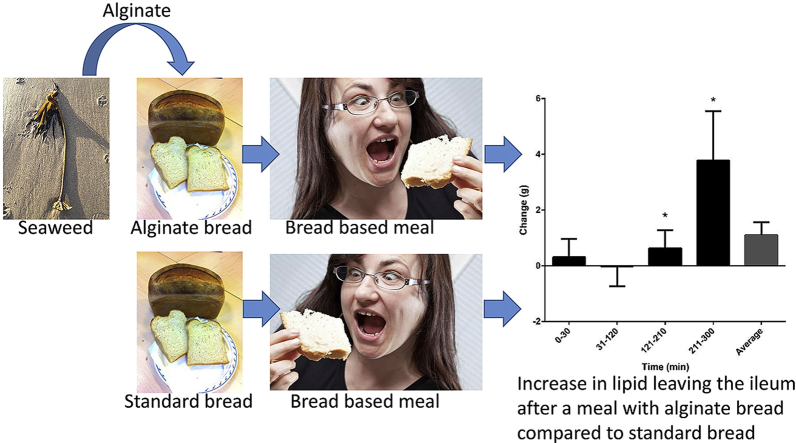

## 1 Introduction

Obesity is defined as an excess of body fat, which results from an imbalance between energy intake and energy expenditure (Trenell, 2015). The incidence of obesity is reaching epidemic proportions worldwide (Galani & Schneider, 2007) and the condition is associated with an increased risk of morbidity and mortality (Fontaine, Redden, Wang, Westfall, & Allison, 2003). Currently there are limited approved pharmacological therapies for managing obesity, and those that are available are associated with negative gastrointestinal side effects, such as; oily spotting on underwear, flatulence, urgent bowel movements, fatty or oily stools, increased number of bowel movements, inability to control bowel movements, gas with discharge, and loose stools (Daneschvar, Aronson, & Smetana, 2016; Kang & Park, 2012). Although there are a number of promising agents currently being assessed (Hardy, Anstee, & Day, 2015) such as gut microbiome transplantations (Jayasinghe, Chiavaroli, Holland, Cutfield, & O'Sullivan, 2016) and Korean Mistletoe extract (Jung et al., 2013), lifestyle interventions incorporating weight loss and physical activity/exercise remain the cornerstone of obesity management (Galani & Schneider, 2007; Thoma, Day, & Trenell, 2012). However, clinical implementation and adherence is difficult (Ayyad & Andersen, 2000).

An alternative approach to reducing the amount of energy consumed is to reduce the absorption of macronutrients once consumed. Fat is the most energy-dense macronutrient, and can account for up to 40% of the energy consumed in a Western diet (Mu & Høy, 2004). Therefore, if the absorption of fat could be reduced once ingested, this would be a valid target as a treatment for obesity (Drent & Vanderveen, 1993; Hadvary, Lengsfeld, & Wolfer, 1988). Pancreatic lipase is responsible for between 70 and 80% of fat digestion (Borel et al., 1994; Carey, Small, & Bliss, 1983), therefore an attenuation of this would reduce the digestion of fat, meaning fat passes through the upper gastrointestinal (GI) tract undigested (Carey et al., 1983; Mu & Høy, 2004). Previous research has shown that tetrahydrolipstatin (orlistat) is able to inhibit pancreatic lipase by covalently binding to its active site (Hadvary et al., 1988; Hadvary, Sidler, Meister, Vetter, & Wolfer, 1991), and the drug has been shown to be effective in weight loss studies (Hollander et al., 1998). However, due to the side effects experienced by patients, compliance with orlistat treatment has been reported to be as low as 47%, even with the intervention of pharmacists (Malone & Alger-Mayer, 2003; Sjostrom et al., 1998). Previous studies have combined the administration of orlistat with dietary fibers such as psyllium husk, to successfully reduce the GI side effects associated with the drug (Cavaliere, Floriano, & Medeiros-Neto, 2001). Therefore a dietary fiber that is able to inhibit pancreatic lipase without the side effects of orlistat may be an effective and viable tool to combat obesity.

We have previously shown that alginate, a dietary fiber extract of brown seaweed, is effective at reducing pancreatic lipase activity *in vitro* (Houghton et al., 2015; Wilcox, Brownlee, Richardson, Dettmar, & Pearson, 2014). We have also shown that alginate can be incorporated into food products, and is released under simulated upper GI tract conditions *in vitro*, where pancreatic lipase is active (Houghton et al., 2014). The primary aim of this study was to assess whether bread with the addition of alginate was able to inhibit fat digestion in ileostomy patients. The secondary aim was to assess the acceptability and potential of GI side effects of longer term consumption of alginate bread on GI wellbeing in healthy volunteers.

## 2 Materials and methods

### 2.1 Materials

Chloroform, methanol and sodium chloride were purchased from Sigma-Aldrich (Poole, UK) Total plasma triglycerides assay kits were purchased from Amsbio (Abingdon, UK). Alginate MANUCOL DM was a gift from FMC BioPolymer (Drammen, Norway). The standard white bread and alginate bread were produced by Greggs Plc (Newcastle upon Tyne, UK), with the alginate bread at 4% alginate (w/w) of wet dough. Manucol DM was used because, when compared to a range of alginate it made the best bread. The rate of alginate inclusion in bread was to create a high fibre bread without seriously affecting the quality of the bread, as determined by Greggs Plc. Manucol DM had a viscosity of 150–300 mPa s (cp) for a 1% solution in deionised water, based on this viscosity measurement an estimated average molecular weight was 250,000. The nutritional profile per 100 g for control bread was; energy (kcal) 247, protein 10.2 g, carbohydrates 46.2 g, fat 1.7 g, fiber 3 g, sodium 0.4 g, and water 36.8 g. The nutritional profile per 100 g for alginate bread was; energy (kcal) 304.5, protein 7.6 g, carbohydrates 41.4 g, fat 13.3 g, fiber 2.8 g, Alginate 4 g, sodium 1.4 g, and water 29.9 g. Based on 15% loss in mass from baking the alginate concentration would have increased to 4.7%. The bakers added the additional fat to ensure that the alginate was evenly distributed within the dough.

### 2.2 Methods

#### 2.2.1 Acceptability study

The assessment of two week consumption of alginate bread was performed with 54 participants (29 females) with a median age of 31 (range of 18–67). The study protocol was approved by the Newcastle University, Faculty of Medical Sciences Ethical Review Process and registered with ClinicalTrials.gov (NCT03477981). All participants were within the Newcastle area, over 18 years old, free from any known chronic illnesses and were not planning any changes in dietary habits, physical activity or in body weight for six months prior to and during the study. Participants were instructed to replace bread they normally consumed with the breads provided by the study team. This study lasted 4 weeks in total and participants were given control bread for the first two weeks (week 1 and week 2), followed by the alginate bread for a further two weeks (week 3 and week 4). All bread was provided upon request by the study team. Participants filled in daily food diaries and completed a visual analogue scale (VAS) wellbeing questionnaire after their main evening meal. The validity, reproducibility and use of VAS has previously been reported (Flint, Raben, Blundell, & Astrup, 2000) and was therefore selected to subjectively assess gastrointestinal wellbeing after consuming alginate and control bread. The VAS used a 100 mm line, which expressed the most positive feelings at one end and the most negative feelings at the other end. A second series of VAS questions were answered at the end of every week. Both questionnaires (asking both sets of questions) were completed 24 h prior to study commencement to provide baseline data. Dietary intake was assessed across both treatments using 7 day unweighted food diaries completed each week of the intervention, with portion size estimates coming from nationally (UK) representative data (Agency, 2014). These data were subsequently processed by an independent researcher blinded to the study design for energy macronutrient and dietary fiber intakes, with outputs from the dietary analysis software NetWISP (version 3.0 for Windows, Tinuviel Software, Warrington, UK). All 54 participants completed both treatment arms across the four weeks for the bread acceptability study.

#### 2.2.2 Fat digestion study

The fat digestion study was a double-blind, randomised, controlled cross-over pilot study (with all randomisation by an independent researcher) which assessed the acute physiological impact of a single high-fat test meal containing alginate. The study protocol was approved by the County Durham & Tees Valley Research Ethics Committee, UK (10/H0908/44) and all participants gave written informed consent. The study was registered with ClinicalTrials.gov (NCT03350958).

Participants were recruited from areas around Newcastle Upon Tyne, UK, through advertising and attending gastroenterology and ileostomy meetings. Inclusion criteria were: non-smoking, 18 years and over, have a well-functioning and stable ileostomy for at least 2 years, have not been pregnant in the past twelve months or planning to be, and not planning to change dietary habits or levels of physical activity during the study. Twenty nine volunteers completed the study (17 females), BMI of 27.4 (±0.02) with a median age of 61 (range 32–83) with 28 volunteers giving blood samples.

Participants underwent telephone screening before being invited to attend the research facility for an induction visit. Participants were provided with two identical meals and two 500 mL bottles of water to take home with them. Participants were instructed to consume one meal and one bottle of water on the evening prior to their two visits to the study centre (plus any additional water *ad libitum*). Participants were randomly allocated to one of two treatment groups, Group A or Group B by an independent researcher. Group A received 100 g of alginate bread, as toast, with 20 g of butter, whereas Group B received 100 g of control bread, as toast, with 20 g of butter. The treatments were reversed for the second visit. Participants were instructed to fast for at least 12 h prior to arriving at the study centre for each visit. Upon arrival, ileal effluent and fasting blood samples were collected and wellbeing questionnaires were filled out, participants were then provided with their test meal and water. Blood samples, effluent samples and wellbeing questionnaires were collected every 30 min following consumption of the test meal. Effluent was weighed and stored at −20 °C along with plasma samples until further analysis. Participants returned to the study centre two to four weeks later and the second acute meal study was performed. Total lipid extraction of ileal effluent was performed as previously described (Folch, Lees, & Sloane Stanley, 1957) and total plasma triglycerides were measured. Seven participants originally recruited did not complete the fat digestion study due to personal time constraints, not because of any aspects of the experimental protocol. Thus data are presented for twenty-nine participants who completed the fat digestion study. Of these only 26 volunteers produced sufficient ileal effluent for measurements and only 28 volunteers provided blood samples.

### 2.3 Statistical analysis

Data from the acceptability study VAS were analysed by one way ANOVA with Kruskal-Wallis multiple comparison test. Energy, dietary fiber (as non-starch polysaccharide) and macronutrient data were analysed by Wilcoxon matched pairs signed rank test. A paired *t*-test was used to test total plasma triglycerides, effluent weights, and total lipid content. Data are presented as the mean with standard error of the mean (±S.E.M.) Statistical significance was set at p ≤ 0.05. Statistical analyses were performed using Graphpad Prism software (Version 6, La Jolla, CA, USA).

## 4 Results

### 4.1 Bread acceptability study

In the daily wellbeing questionnaire, participants felt significantly more alert when consuming alginate bread compared with control bread (week 1) during weeks 3 and 4. Participants also felt calmer and more relaxed, when consuming alginate bread (week 4 only) compared with control bread (week 1 only) (Table 1). Participants were significantly fuller when consuming the alginate bread compared with baseline and week 1 but there were no differences in feelings of nausea, bloatedness or flatulence during the four weeks on either treatment.

**Table 1:** Mean (±SD) visual analogue scale (VAS) of the wellbeing questions asked of volunteers at the end of every day.

0 – 10	Baseline	Week 1	Week 2	Week 3	Week 4
Alert – Sleepy	3.3 ± 2.1^a,b^	3.8 ± 2.6^a^	3.4 ± 2.7^a,b^	3.1 ± 2.5^b^	3.0 ± 2.6^b^
Fine – Nauseous	1.1 ± 1.1^a^	2.0 ± 2.2^a^	1.8 ± 2.0^a^	2.0 ± 2.1^a^	1.8 ± 2.1^a^
Full – Starving	3.7 ± 1.8^a^	3.0 ± 2.1^a,b^	2.8 ± 2.0^b,c^	2.5 ± 1.9^c^	2.5 ± 2.0^c^
Not Bloated – Bloated	2.1 ± 2.0^a^	2.7 ± 2.7^a^	3.0 ± 2.8^a^	3.0 ± 2.6^a^	2.6 ± 2.5^a^
Not Flatulent – Flatulent	2.5 ± 2.6^a^	2.7 ± 2.5^a^	2.9 ± 2.5^a^	3.0 ± 2.6^a^	2.8 ± 2.6^a^
Calm – Irritable	2.2 ± 2.1^a,b^	2.6 ± 2.2^a^	2.4 ± 2.1^a,b^	2.3 ± 2.2^a,b^	2.1 ± 2.0^b^
Relaxed – Anxious	2.4 ± 2.1^a,b^	2.7 ± 2.2^a^	2.4 ± 2.1^a,b^	2.4 ± 2.2^a,b^	2.2 ± 2.0^b^

Baseline data was taken the day before the volunteers began the study. Week 1 and week 2 the volunteers only consumed the standard white bread, week 3 and week 4 volunteers only consumed alginate bread. Within each row, averages with the same letter are not statistically different based on analysis with a one way ANOVA with Kruskal-Wallis multiple comparison test.

Weekly questions were also very similar between the alginate and control bread over the duration of the study (Table 2). There was, however, a significant difference between baseline and week 4 where participants had an increase in ‘light-headedness or dizziness when consuming alginate bread (*P* < 0.05) (Table 2). Similarly there was also an increase at week 4 (alginate bread) of ‘blurred vision’ compared with baseline. There was also a statistical increase in abdominal discomfort, between baseline and weeks 2 (control bread), 3, and 4 (alginate bread), however there were no differences between the weeks when either bread was consumed. Abdominal pain or discomfort was also significantly higher during the measurement period compared with baseline. There was also a significant increase in flatulence between baseline and week 3, the first week consuming alginate bread although the differences were very small (a change of 0.6 on the VAS).

**Table 2:** Mean (±SD) visual analogue scale (VAS) of the wellbeing questions asked of volunteers at the end of week.

0 – 10	Baseline	Week 1	Week 2	Week 3	Week 4
**Light-headedness or dizziness?** Not at all – Very	0.7 ± 1.4^a^	1.2 ± 2.1^a,b^	0.9 ± 1.2^a,b^	1.4 ± 1.9^a,b^	1.3 ± 1.4^b^
**Blurred Vision?** Not at all – Very	0.4 ± 0.6^a^	0.5 ± 1.0^a,b^	0.6 ± 0.8^a,b^	0.8 ± 2.1^a,b^	1.0 ± 1.3^b^
**A difficulty to concentrate?** Not at all – Very	1.3 ± 1.7^a^	1.5 ± 1.7^a^	1.6 ± 1.8^a^	1.5 ± 1.8^a^	1.8 ± 2.0^a^
**A difficulty to think?** Not at all – Very	1.2 ± 1.5^a^	1.4 ± 1.6^a^	1.5 ± 1.6^a^	1.6 ± 1.9^a^	1.5 ± 1.6^a^
**Excessive thirst?** Not at all – Very	1.3 ± 1.8^a^	1.4 ± 1.7^a^	1.4 ± 1.4^a^	1.6 ± 1.6^a^	1.9 ± 2.0^a^
**Headaches/migraines?** Not at all – Very	1.3 ± 2.0^a^	2.0 ± 2.5^a^	1.6 ± 2.1^a^	1.7 ± 2.1^a^	2.0 ± 2.4^a^
**Craving for sweets?** Not at all – Very	2.4 ± 2.4^a^	2.2 ± 2.5^a^	2.6 ± 2.4^a^	2.1 ± 2.2^a^	2.5 ± 2.6^a^
**Abdominal discomfort?** Not at all – Very	0.7 ± 0.9^a^	1.9 ± 2.4^a,b^	2.1 ± 2.3^b^	2.0 ± 2.2^b^	2.0 ± 2.3^b^
**Bowel Habit?** Constipated – Diarrhoea	4.5 ± 0.8^a^	4.8 ± 1.3^a^	4.6 ± 1.2^a^	4.2 ± 1.3^a^	4.7 ± 1.4^a^
**Urgency to pass stool?** Less than norm – More than norm	4.4 ± 0.9^a^	4.6 ± 1.3^a^	4.8 ± 1.0^a^	4.8 ± 1.0^a^	4.8 ± 1.3^a^
**Abdominal pain or discomfort?** No pain – Terrible	1.7 ± 2.1^a^	2.6 ± 2.4^a,b^	3.0 ± 2.5^b^	2.7 ± 2.3^a,b^	3.2 ± 2.4^b^
**Amount of flatulence?** Less than norm – More than norm	4.6 ± 1.2^a^	4.6 ± 1.6^a,b^	4.6 ± 1.6^a,b^	5.2 ± 1.3^b^	5.0 ± 1.7^a,b^

Baseline data was taken the day before the volunteers began the study. Week 1 and week 2 the volunteers only consumed the standard white bread, week 3 and week 4 volunteers only consumed alginate bread. Within each row averages with the same letter are not statistically different based on analysis with a one way ANOVA with Kruskal-Wallis multiple comparison test.

Total dietary intakes of energy, carbohydrate and protein were not significantly different between the periods consuming control and alginate breads (*P* > 0.05) (Table 3). Daily fat intake was significantly higher during the alginate bread period although the median values were very similar. The intake of non-starch polysaccharides (NSP) was significantly increased by about 2 g/d as expected when the volunteers were consuming alginate bread compared with the standard white bread and the percentage of NSP from bread increased significantly from 13 to 29%.

**Table 3:** Dietary intake data across the two treatment groups.

Treatment	Standard (median (IQR))	Alginate (median (IQR))	*P*-value
Energy intake (MJ/d)	7.4 (6.5–8.9)	7.7 (5.8–8.8)	0.164
CHO intake (g/d)	198.7 (168.2–240.4)	200.2 (168.3–246.9)	0.707
Protein intake (g/d)	75 (66.6–85.2)	74.7 (65.6–81.6)	0.527
Fat intake (g/d)	68.8 (55.1–79.9)	68.9 (49.9–80.6)	0.012
NSP intake (g/d)	11 (8.2–13.0)	12.8 (10.7–15.8)	<0.001
Slices of bread (/d)	1.8 (1.4–2.3)	1.8 (1.4–2.2)	0.543
% NSP from bread	12.9 (9.9–17.5)	28.5 (23.9–35.8)	<0.001

All values represent medians (Inter Quartile Range), CHO – carbohydrate, NSP – Non-starch polysaccharide.

### 4.2 Fat digestion

**Fig. 1:**
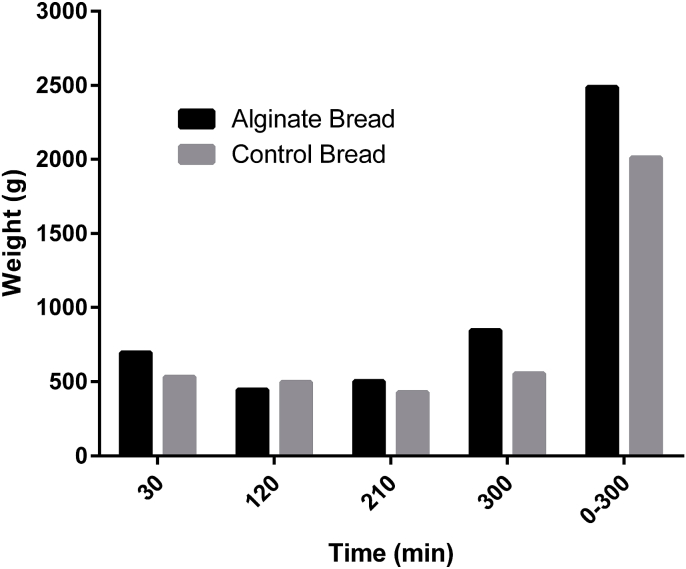
wt of ileal effluent following consumption of alginate bread and control bread. The weight of effluents were combined into four time periods; up to 30 min, 31–120 min, 121–210 min and 211–300 min, as well as a total weight for each treatment. There are no error bars as the ileal effluent for all volunteers was combined.

The ileal effluent from the volunteers was weighed and grouped into four time periods; 0–30, 31–120, 121–210 and 211–300 min, and combined to calculate total effluent weight for alginate and control bread (Fig. 1). The weight of the effluent increased with consumption of alginate bread in three out of the four time periods, with the largest increase (291 g) occurring between 211 and 300 min. Cumulatively, an increase in weight of 478 g of ileal effluent was observed when the participants consumed alginate bread.

**Fig. 2:**
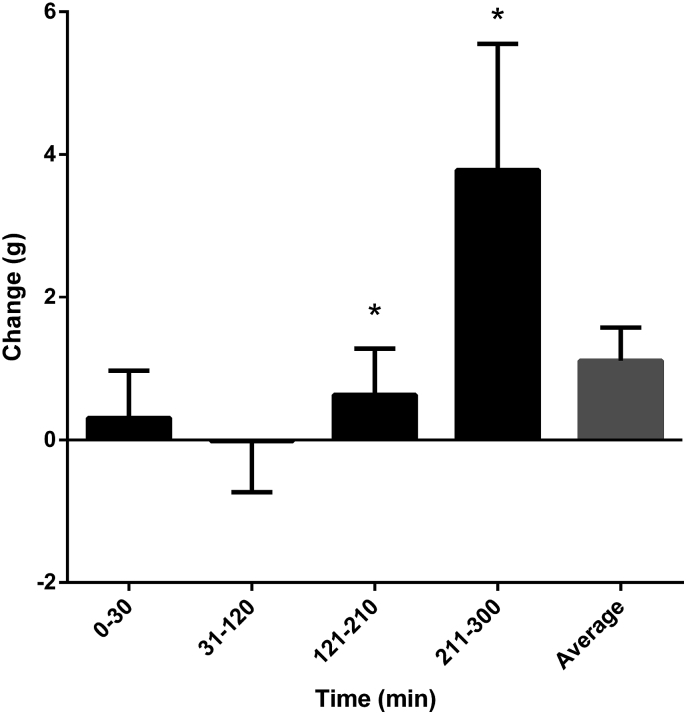
The change in weight of lipid from consumption of control bread to alginate bread. The weight of lipid in the effluent after consuming control bread was subtracted from that of consuming alginate bread for each volunteer. The figure shows the mean and standard error of each time point (up to 30, 31–120, 121–210, and 211–300 min) as well as the average change in lipid content over the total time period tested. * Indicate values significantly different to values at 31-120 min.

The lipid content recovered in effluent fluid was similar for alginate and control bread from 0 to 30 and 31–120 min (Fig. 2). However, there was a significant increase in lipid content in the effluent from 121 to 210 to 211–300 min after consumption of alginate bread (3.8 g ± 1.6). Overall there was an average increase of 1.1 g ± 0.5 of lipid content in the ileal effluent when volunteers consumed alginate bread.

**Fig. 3:**
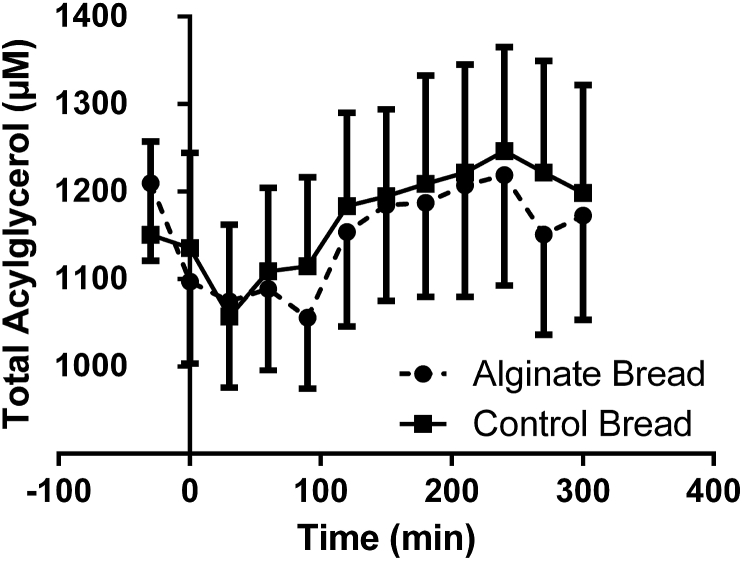
Triacyl, diacyl and monoacylglycerol in plasma samples of volunteers during consumption of the test meal and for 5 h after. Square symbols represent data for control bread and solid circles represent data for alginate bread.

The initial measurements of triacylglycerol in plasma were within expected normal fasting range (Fig. 3). Alginate bread consistently reduced plasma triacylglycerol, although the individual time points were not significantly different when compared with the control bread. A 2% reduction in area under the curve was seen for the concentration of total triacylglycerol in plasma when volunteers consumed alginate bread compared with control bread, although again this was not significant.

## 5 Discussion

This is the first study to demonstrate that alginate enriched bread was well tolerated and capable of attenuating lipid digestion. Alginate enriched bread and control bread were equally well tolerated, with only minor adverse side effects, with no withdrawals during the acceptability study. In the fat digestion study alginate enriched bread increased effluent weight and lipid content in effluent (by an average of 1.1 g ± 0.5) and reduced total plasma triglycerides (by 2% AUC) in ileostomy patients compared with control bread.

The addition of alginate to food or drink has been shown to reduce subsequent food intake after a meal (Holt, Miller, Petocz, & Farmakalidis, 1995) but also reduces palatability (Georg Jensen, Kristensen, Belza, Knudsen, & Astrup, 2012; Vuksan et al., 2009). The reduction in palatability has been suggested to be due to the increase in viscosity alginate gives and therefore, the alteration in mouthfeel (Vuksan et al., 2009). However in this study we did not test the impact of alginate on the orosensory modalities of bread. This increase in viscosity is also likely to play a role in satiety and the reduced food intake due to changes in the rate of stomach emptying (Holt et al., 1995; Paxman, Richardson, Dettmar, & Corfe, 2008). In the present study alginate was added to bread as a functional ingredient to potentially reduce fat digestion, but a significant increase in the feeling of fullness was also observed when consuming alginate bread (compared with baseline data and the first week of control bread), confirming previous studies that alginates increase satiety, decrease hunger and reduce energy intake (Hoad et al., 2004; Jensen, Knudsen, Viereck, Kristensen, & Astrup, 2012a; Paxman et al., 2008). Participants reported an increase in alertness and reduced anxiety (in the daily questionnaire) when consuming alginate bread compared with the first week of the study. This may be due to potential apprehension of the initiation of the trial, a new experience for many of the volunteers. However, these data could be explained, in part, by a bias of the subjects being able to differentiate between the breads.

In the weekly questionnaire there was an increase in abdominal discomfort and abdominal pain for both the alginate and control bread, although the difference was small. Previous studies using alginate food products have not reported such findings (Georg Jensen, Kristensen, & Astrup, 2011; Georg Jensen et al., 2012) and with the same increase seen in the control bread, this may be due to the increase in consumption of bread over their habitual levels. There was an increase in the level of perceived flatulence during the first week consuming the alginate bread. An increase in the consumption of NSP (1.8 g per day) when consuming alginate bread may be a possible explanation for the increase in flatulence as more will enter the colon and be processed by the microflora present. Continued ingestion of NSP increases the availability of macronutrient, energy, and carbon sources to the microbial community and would be expected to alter the microflora over time (Brownlee et al., 2005; Scott, Gratz, Sheridan, Flint, & Duncan, 2013) potentially reducing the level of flatulence. This increase in NSP intake observed with alginate bread would also be beneficial in helping people achieve the recommended intake of fiber (Howarth, Saltzman, & Roberts, 2001), improve GI motility and maintain a healthy GI system (Holt et al., 1995).

Increased fiber intake has been linked with modulating appetite, as demonstrated in the current study with an increase in the feeling of fullness (Kristensen & Jensen, 2011). Previous studies have reported reduced daily energy consumption by 7% (Paxman et al., 2008) which may be associated with weight loss in previous studies (Georg Jensen et al., 2012). However in the current study there was no significant difference in total energy intake between the control and alginate bread consumption periods. The delivery vehicle will play a large role on the effect of satiety with both of the aforementioned studies (Jensen et al., 2012b; Paxman et al., 2008) using a drink to deliver the alginate. When added to a drink alginate forms a viscous solution or gel in the stomach, increasing the volume and activating stretch receptors. This increased bolus in the stomach may therefore account for increased feeling of fullness and reduced energy intake. Alginate cooked into bread is unlikely to have the same effect and our *in vitro* data suggest that the alginate may not actually be released from the bread matrix until it reaches the duodenum (Houghton et al., 2014). Houghton et al. (2014) showed a small release of alginate in the gastric phase from the bread giving a concentration of around 0.1 mg/ml, below a gelling concentration (Houghton et al., 2014). In addition Houghton et al. (2015) has shown that baking produces alginate fragments which would also reduce its gelling potential (Houghton et al., 2015).

Unsurprisingly, NSP intake appeared to increase when participants consumed the alginate bread compared with the standard bread by approximately 3 g per day (see Table 1). It must be noted that overall fiber intake was low within the participants and this increase in intake still meant that only 1 out of 54 participants met their recommended dietary allowance of NSP intakes of 24 g per day or above (England, 2015). This increase would equate to a relatively modest consumption of the study bread, which provided approximately an extra 2 g of fiber per slice compared with the control bread. Intake of bread ranged from as low 0.6 slices a day to almost eight slices a day among the participants, although the intake between the two treatment groups was not statistically different.

Alginate has previously been shown to inhibit pancreatic lipase activity (Houghton et al., 2015; Wilcox et al., 2014). Any reduction in activity would ultimately result in a reduction in fat digestion and absorption. In the current study, there was a 24% increase in effluent weight and 40% increase of fat content in effluent fluid when participants consumed alginate bread. It is possible that some of the fat in the ileal effluent for the alginate enriched bread arm of the study may have in some part come from the extra fat in the bread. This may result, as it may not be easily digested, due to colloidal changes occurring e.g the alginate forming an acid gel trapping the fat. This can be ruled out based on the work of Houghton et al. (2014) showing the alginate is retained in the bread matrix and is not released until the higher pH of the small intestine (Houghton et al., 2014). Ileostomists have been used to demonstrate changes in ileal effluent lipid content before with consumption of other fibers (Bosaeus, Carlsson, Sandberg, & Andersson, 1986; Higham & Read, 1992) but also with alginate (Sandberg et al., 1994b). The small, six volunteer, study with alginate used milkshakes as the vehicle but only measured free fatty acids – the breakdown product of lipase activity (Sandberg et al., 1994b). However, they did show an increase in the amount of free fatty acids leaving the small intestine, potentially showing a higher level of lipase activity, contrary to that proposed in this study. Alginate may also reduce transit time by adding to the luminal bulk (Brownlee et al., 2010), which has been linked with the water binding capacity of fiber (Chaplin, 2003; Sandberg et al., 1994a). This may, in part, explain the increased effluent weight when participants consumed alginate bread. Alginate, which should pass through the upper GI relatively undigested, may contribute to the increased effluent weight reported here. A dry matter measurement would have addressed this point but unfortunately was not performed. Participants consumed alginate bread which included 4 g of alginate each, totalling 104 g (4 × 26) but this does not account for the difference in total effluent weight (478 g) between the two arms. These data suggest that alginate bread is able to increase effluent weight possibly through water retention and increase fat recovered in effluent, potentially by inhibiting pancreatic lipase. This suggests that alginate-enriched bread has potential as an obesity treatment and should be explored further.

Any inhibition or delay of macronutrient digestion in the upper GI tract will ultimately be reflected in the circulatory system. Previous studies have observed reduction in blood glucose and insulin concentrations following the addition of alginate to a drink (Torsdottir, Alpsten, Holm, Sandberg, & Tolli, 1991) and a cereal bar (Williams et al., 2004). In the current study, the primary aim was to investigate whether alginate added to bread was able to reduce fat digestion and potentially plasma triglycerides. The reduction in plasma triacylglycerol supports the additional effluent weight and the increase in total lipid recovered in effluent. The potential mechanism for this is the reduced enzymatic activity of lipase in the small intestine that limits the amount and rate of release of fatty acids, monoglycerides and glycerol from the TAG in a meal, consequently reducing the rate of TAG resynthesis in the enterocytes. The fat content of the two breads were not identical, alginate bread contained 13.3 g/100 g compared with 1.7 g/100 g for the control. Despite more fat in the alginate bread the plasma triglyceride concentrations appeared to be lower when participants consumed alginate bread, suggesting that alginate may have attenuated fat absorption. The level of pancreatic lipase released into the GI tract would be far in excess of what would be needed to digest the fat present in the meal given; therefore even if more fat was consumed, normal pancreatic activity should be able to digest all the fat.

## 6 Conclusion

Incorporation of specific alginates in bread appears to limit the amount of fat that can be absorbed from a single meal. Further long-term studies are required to see if these findings translate to a positive impact on body weight or cardiovascular health.

## Funding

This work was funded by the BBSRC (grant number BB/G00563X/1).
